# Maternal Tryptophan Supplementation Protects Adult Rat Offspring against Hypertension Programmed by Maternal Chronic Kidney Disease: Implication of Tryptophan-Metabolizing Microbiome and Aryl Hydrocarbon Receptor

**DOI:** 10.3390/ijms21124552

**Published:** 2020-06-26

**Authors:** Chien-Ning Hsu, I-Chun Lin, Hong-Ren Yu, Li-Tung Huang, Mao-Meng Tiao, You-Lin Tain

**Affiliations:** 1 Department of Pharmacy, Kaohsiung Chang Gung Memorial Hospital, Kaohsiung 833, Taiwan; cnhsu@cgmh.org.tw; 2 School of Pharmacy, Kaohsiung Medical University, Kaohsiung 807, Taiwan; 3 Department of Pediatrics, Kaohsiung Chang Gung Memorial Hospital and Chang Gung University College of Medicine, Kaohsiung 833, Taiwan; uc22@adm.cgmh.org.tw (I.-C.L.); yuu2002@cgmh.org.tw (H.-R.Y.); huangli@cgmh.org.tw (L.-T.H.); tmm@cgmh.org.tw (M.-M.T.); 4 Department of Medicine, Chang Gung University, Linkow 244, Taiwan; 5 Institute for Translational Research in Biomedicine, Kaohsiung Chang Gung Memorial Hospital and Chang Gung University College of Medicine, Kaohsiung 833, Taiwan

**Keywords:** aryl hydrocarbon receptor, chronic kidney disease, developmental origins of health and disease (DOHaD), gut microbiota, hypertension, nitric oxide, renin–angiotensin system, tryptophan, pregnancy

## Abstract

Hypertension and chronic kidney disease (CKD) can originate during early-life. Tryptophan metabolites generated by different pathways have both detrimental and beneficial effects. In CKD, uremic toxins from the tryptophan-generating metabolites are endogenous ligands of the aryl hydrocarbon receptor (AHR). The interplay between AHR, nitric oxide (NO), the renin–angiotensin system (RAS), and gut microbiota is involved in the development of hypertension. We examined whether tryptophan supplementation in pregnancy can prevent hypertension and kidney disease programmed by maternal CKD in adult offspring via the aforementioned mechanisms. Sprague–Dawley (SD) female rats received regular chow or chow supplemented with 0.5% adenine for 3 weeks to induce CKD before pregnancy. Pregnant controls or CKD rats received vehicle or tryptophan 200 mg/kg per day via oral gavage during pregnancy. Male offspring were divided into four groups (*n* = 8/group): control, CKD, tryptophan supplementation (Trp), and CKD plus tryptophan supplementation (CKDTrp). All rats were sacrificed at the age of 12 weeks. We found maternal CKD induced hypertension in adult offspring, which tryptophan supplementation prevented. Maternal CKD-induced hypertension is related to impaired NO bioavailability and non-classical RAS axis. Maternal CKD and tryptophan supplementation differentially shaped distinct gut microbiota profile in adult offspring. The protective effect of tryptophan supplementation against maternal CKD-induced programmed hypertension is relevant to alterations to several tryptophan-metabolizing microbes and AHR signaling pathway. Our findings support interplay among tryptophan-metabolizing microbiome, AHR, NO, and the RAS in hypertension of developmental origins. Furthermore, tryptophan supplementation in pregnancy could be a potential approach to prevent hypertension programmed by maternal CKD.

## 1. Introduction

Hypertension and kidney disease are highly prevalent diseases throughout the world. Hypertension is both a cause and result of kidney disease. Emerging evidence suggests that the origins of both hypertension and kidney disease can originate in early life [1,2,3]. Maternal illness and nutrition play key roles in the developmental programming of kidney disease and hypertension [1,2,3]. Although women with chronic kidney disease (CKD) are at risk for adverse pregnancy-related events [4], less attention has been paid to explore the effects of maternal CKD on the adult offspring outcomes.

Tryptophan is an essential amino acid, which must be supplied in the diet. A plethora of tryptophan-generating metabolites act on the metabolic crossroad interconnecting different organs and have both detrimental and beneficial effects [5,6,7,8]. Endogenous tryptophan metabolites (e.g., melatonin, serotonin, and kynurenines) and gut microbiota derived tryptophan metabolites (e.g., indole, indolic acid, skatole, and tryptamine) play significant roles in developing health and disease. Although dietary tryptophan supplementation has shown potential benefits to the therapy of several human disorders [7,8], so far, no data supported the notion that additional tryptophan in pregnancy could be beneficial for adult offspring outcomes.

In CKD, uremic toxins from the kynurenine and indole pathway are endogenous ligands of the aryl hydrocarbon receptor (AHR) [9]. The AHR can activate distinct transcription factor pathways that contributes to hypertension [8]. We previously reported that prenatal exposure to AHR ligand, like 2,3,7,8-tetrachlorodibenzo-p-dioxin (TCDD) or bisphenol A, induced programmed hypertension in adult offspring [10,11]. The AHR pathway has been reported to interact with nitric oxide (NO) [10,11,12], the renin–angiotensin system (RAS) [13], and gut microbiota [14], which are crucial mechanisms underlying hypertension, to affect blood pressure (BP). However, whether the above-mentioned mechanisms interrelate to maternal CKD-induced offspring hypertension and kidney disease of developmental origins remain unclear.

The adenine-induced CKD rat is a model of progressive kidney injury with cardiovascular changes, mimicking closely the pathophysiology of human CKD [15]. Our aim in this study was first to determine whether maternal adenine-induced CKD causing programmed hypertension in adult offspring is related to altered gut microbiota, reduced NO bioavailability, dysregulated AHR pathway, and imbalanced RAS. The second aim was to determine whether maternal tryptophan supplementation can be a therapeutic approach to prevent hypertension programmed by maternal CKD in adult offspring.

## 2. Results

### 2.1. Blood Pressure and Renal Function

**Figure 1:**
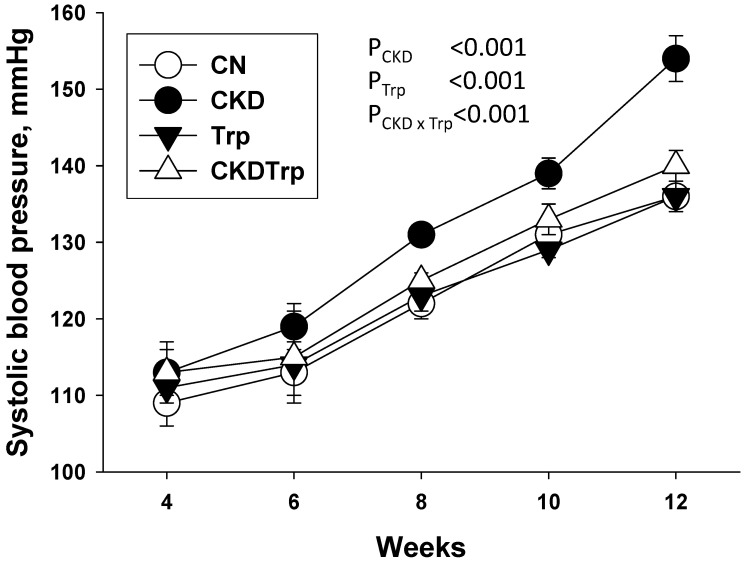
Effects of maternal adenine-induced chronic kidney disease (CKD) and tryptophan supplementation (Trp) on systolic blood pressure measured in male offspring at 4, 6, 8, 10, and 12 weeks of age. Data are shown as means ± S.E.M. A two-way ANOVA was performed for statistical analysis. CKD × Trp = interaction of CKD and Trp. *n* = 8/group.

Adenine-treated female rats had a lower body weight (BW; 211 ± 5 vs. 243 ± 8 g), but a higher kidney weight (2.9 ± 0.17 vs. 1.29 ± 0.04 g), kidney weight-to-BW ratio (1.37 ± 0.06 vs. 0.53 ± 0.01), creatinine level (28.9 ± 7.6 vs. 18.9 ± 0.8 μM), and systolic BP (SBP; 147 ± 1 vs. 127 ± 1 mmHg) compared to the controls (*n* = 5/group, all *p* < 0.05). These data indicate that adenine-induced CKD in female rats before pregnancy, which is characterized by reduced renal function (~35%), renal hypertrophy, BW loss, and hypertension. Table 1 shows that no pups were dead in any of the groups. There was little measurable effect of either maternal CKD or tryptophan supplementation on BW. Maternal CKD and tryptophan supplementation are associated with a higher kidney weight-to-BW ratio (PCKD = 0.004 and PTrp = 0.01), while there was no synergistic effect. As shown in Figure 1, longitudinal measurement of SBP from four to 12 weeks of age showed that maternal CKD increased SBP in male offspring (P_CKD_ < 0.001) than those in the controls from 8 to 12 weeks of age. The CKD-induced elevation of SBP was restored by tryptophan supplementation in the CKDTrp group than those in the CKD group. The diastolic BP was comparable among the four groups. Maternal CKD caused a higher mean arterial pressure in the CKD group vs. the controls. Additionally, Table 1 shows that tryptophan supplementation resulted in a lower creatinine level compared to the controls (P_Trp_ = 0.001).

### 2.2. Nitric Oxide Pathway

Since NO deficiency plays a major role in programmed hypertension and kidney disease [2,16], we, hence, investigated whether maternal CKD induced hypertension is related to impaired NO pathway. Table 2 shows that plasma level of l-citrulline, a precursor of l-arginine, was lower in tryptophan-exposed groups (PTrp = 0.011). Either maternal CKD or tryptophan supplementation altered plasma l-arginine level. While only maternal CKD had an effect to increase plasma asymmetric dimethylarginine (ADMA, an endogenous inhibitor of NO synthase) level in the CKD and the CKDTrp groups (P_CKD_ < 0.001). Maternal tryptophan supplementation caused a lower plasma symmetric dimethylarginine (SDMA), an indirect inhibitor of NO synthase, level in the Trp group compared to the controls. Additionally, maternal CKD induced a decrease of l-arginine-to-ADMA ratio (P_CKD_ < 0.001), a marker representing NO bioavailability, which was not affected by tryptophan treatment. Taken together, our findings indicate that maternal CKD and tryptophan supplementation have opposite effect on NO pathway. Maternal CKD-induced hypertension in adult offspring is related to NO deficiency represented by increased plasma ADMA level and decreased l-arginine-to-ADMA ratio. Conversely, the beneficial effect of tryptophan on the elevation of BP could be relevant to decreased plasma SDMA and l-citrulline level.

### 2.3. Aryl Hydrocarbon Receptor (AHR) Pathway

**Figure 2:**
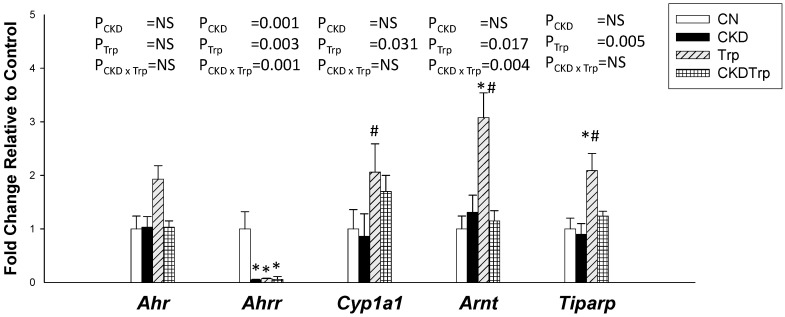
Effects of maternal adenine-induced chronic kidney disease (CKD) and tryptophan supplementation (Trp) on aryl hydrocarbon receptor (AHR) pathway in offspring kidneys at 12 weeks of age. Data are shown as means ± S.E.M. A two-way ANOVA with a Tukey’s post hoc test was performed for statistical analysis. CKD × Trp = interaction of CKD and Trp. * *p* < 0.05 vs. CN. # *p* < 0.05 vs. CKD. *n* = 8/group.

We next measured the expression of the AHR and AHR target genes. The renal *Ahr* mRNA expression was comparable among the four groups (Figure 2). Tryptophan supplementation significantly decreased expression of *Ahrr*, but increased expression of *Cyp1a1*, *Arnt*, and *Tiparp*. Maternal CKD had a negligible effect on most AHR target genes, except there was a lower renal mRNA expression of *Ahrr* in the CKD and CKDTrp groups vs. controls. Additionally, the increases of Trp-induced *Arnt* expression were restored by maternal CKD exposure.

### 2.4. Renin-Antiotensin System

**Figure 3:**
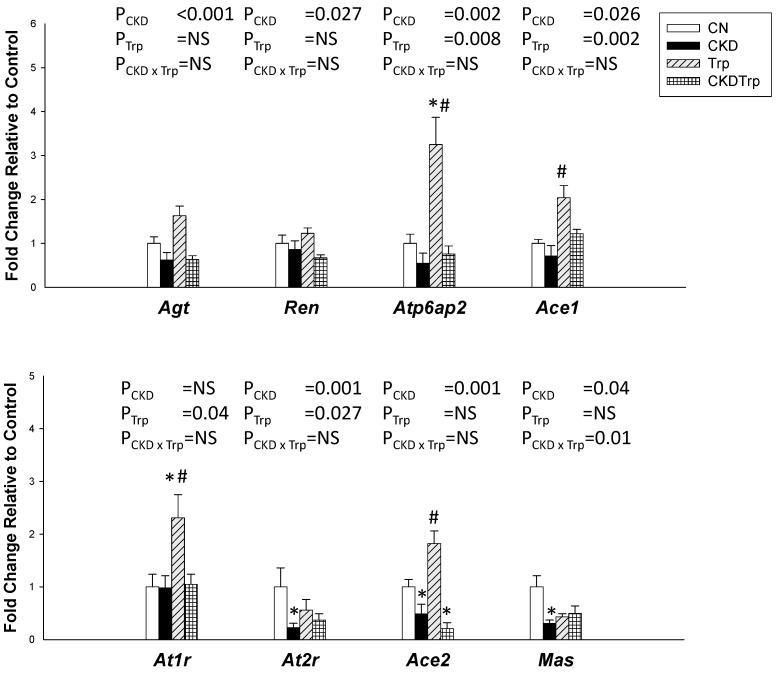
Effects of maternal adenine-induced chronic kidney disease (CKD) and tryptophan supplementation (Trp) on the renin-angiotensin system in offspring kidneys at 12 weeks of age. Data are shown as means ± S.E.M. A two-way ANOVA with a Tukey’s post hoc test was performed for statistical analysis. CKD × Trp = interaction of CKD and Trp. * *p* < 0.05 vs. CN. # *p* < 0.05 vs. CKD. *n* = 8/group.

We further evaluated the renal expression of the RAS components (Figure 3). This system consists of different angiotensin peptides mediated by distinct receptors. The RAS cascade starts with the conversion of angiotensinogen to angiotensin (Ang) I by the renin. The classic RAS, defined as the angiotensin converting enzyme (ACE)-Ang II-angiotensin type 1 receptor (AT1R) axis, promotes sodium retention and elevation of BP. On the contrary, the non-classical RAS composed of the ACE2-Ang-(1-7)-Mas receptor axis leads to vasodilatation [17]. Maternal CKD decreased renal mRNA expression of *Agt* (angiotensinogen), *Ren* (renin), *atp6ap2* (prorenin receptor), *Ace1*, *At2r* (angiotensin type 2 receptor), *Ace2*, and *Mas* (Ang-(1-7) receptor). However, renal mRNA expression of *atp6ap2*, *Ace1*, *At1r*, and *At2r* was higher in the tryptophan-exposed offspring than in the controls.

### 2.5. Gut Microbiota Compositions

**Figure 4:**
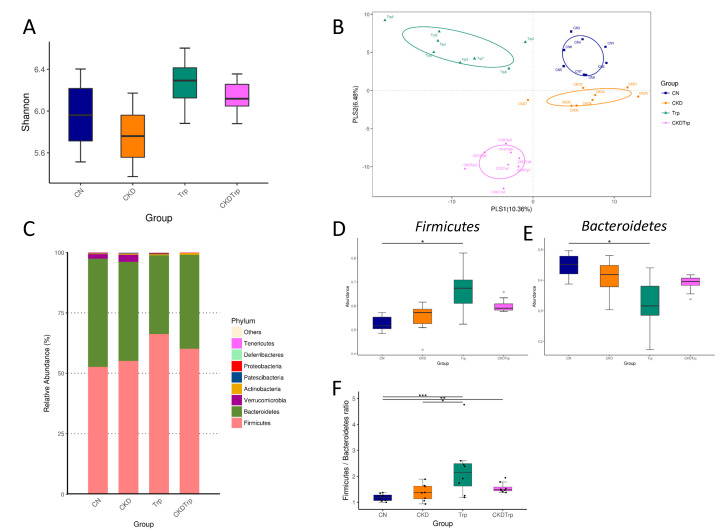
Effects of maternal adenine-induced chronic kidney disease (CKD) and tryptophan supplementation (Trp) on the gut microbiota in offspring at 12 weeks of age. (**A**) Variation in fecal bacterial α-diversity represented by the Shannon’s diversity indexes. (**B**) β-diversity changes in gut microbiota across groups by the Partial Least Squares Discriminant Analysis (PLS-DA). (**C**) Relative abundance of top 10 phyla of the gut microbiota among the four groups. (**D**) Relative abundances of the phylum *Firmicutes*. (**E**) Relative abundances of the phylum *Bacteroidetes*. (**F**) The *Firmicutes* to *Bacteroidetes* ratio. * *p* < 0.05. ** *p* < 0.01. *** *p* < 0.001.

Further, we compared the difference in gut microbiota among the four groups at 12 weeks of age. Microbiome diversity is typically defined in terms of within (i.e., α-diversity) and between community/sample (i.e., β-diversity) diversities [18,19]. The Shannon diversity index is a commonly used α-diversity measure to determine how evenly the microbes are distributed in a community/sample [20]. We found that the Shannon diversity index was not statistically different among the four groups at 12 weeks of age (Figure 4A). We next performed two different β-diversity analysis techniques to compare the bacterial community similarity using the Partial Least Squares Discriminant Analysis (PLS-DA) and the Analysis of Similarities (ANOSIM) [18,19]. Ordination techniques, such as PLS-DA, reduce the dimensionality of microbiome data sets so that a summary of the beta diversity relationships can be visualized in two-dimensional scatterplots [18,19]. ANOSIM was used to test the significant differences between the groups of samples [21]. The scatterplots of PLS-DA analysis showed that all groups were well separated (Figure 4B), indicating that four groups had distinct enterotypes. ANOSIM analysis showed there to be a significant difference between the four groups (All *p* < 0.01). The major bacteria phyla found in 12-week-old offspring were *Firmicutes, Bacteroidetes, Verrucomicrobia, Actinobacteria,* and *Patescibacteria* (Figure 4C). Maternal tryptophan intake caused a remarkable increase in the phylum *Firmicutes* (*p* = 0.019) (Figure 4D), but a decrease in the *Bacteroidetes* (*p* = 0.015) (Figure 4E). The *Firmicutes* to *Bacteroidetes* ratio has been considered a signature for hypertension [22]. In the current study, the *Firmicutes* to *Bacteroidetes* ratio was higher in the Trp (2.29 ± 0.4, *p* < 0.001) and CKDTrp group (1.56 ± 0.07, *p* = 0.002) compared to that in the control group (1.18 ± 0.05) (Figure 4F).

**Figure 5:**
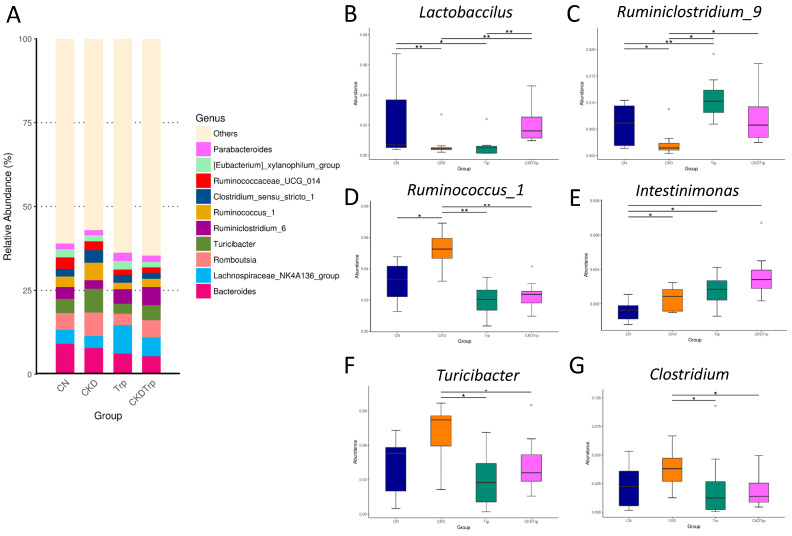
Effects of maternal adenine-induced chronic kidney disease (CKD) and tryptophan supplementation (Trp) on the gut microbiota at the genus level in 12-week-old offspring. (**A**) Relative abundance of top 10 genera of the gut microbiota among the four groups. Relative abundances of the genus (**B**) *Lactobacillus,* (**C**) *Ruminiclostridium_9*, (**D**) *Ruminococcus_1*, (**E**) *Intestinimonas*, (**F**) *Turicibacter,* and (**G**) *Clostridium*. * *p* < 0.05. ** *p* < 0.01. *** *p* < 0.001.

At the genus level, the major genera found in 12-week-old offspring were identical among the four groups (Figure 5A). We observed that maternal CKD decreased the abundance of the genera *Lactobacillus* (Figure 5B, *p* = 0.004) and *Ruminiclostridium_9* (Figure 5C, *p* = 0.02), but increased genus *Ruminococcus_1* (Figure 5D, *p* = 0.02) in the CKD group than those in the controls. These changes were restored by tryptophan supplementation (Figure 5B–D). Compared to the CKD group, tryptophan supplementation significantly increased the abundance of the genus *Intestinimonas* (Figure 5E, *p* = 0.045), while it decreased the abundance of genera *Turicibacter* (Figure 5F, *p* < 0.001) and *Clostridium* (Figure 5G, *p* = 0.035) in the CKDTrp group.

**Figure 6:**
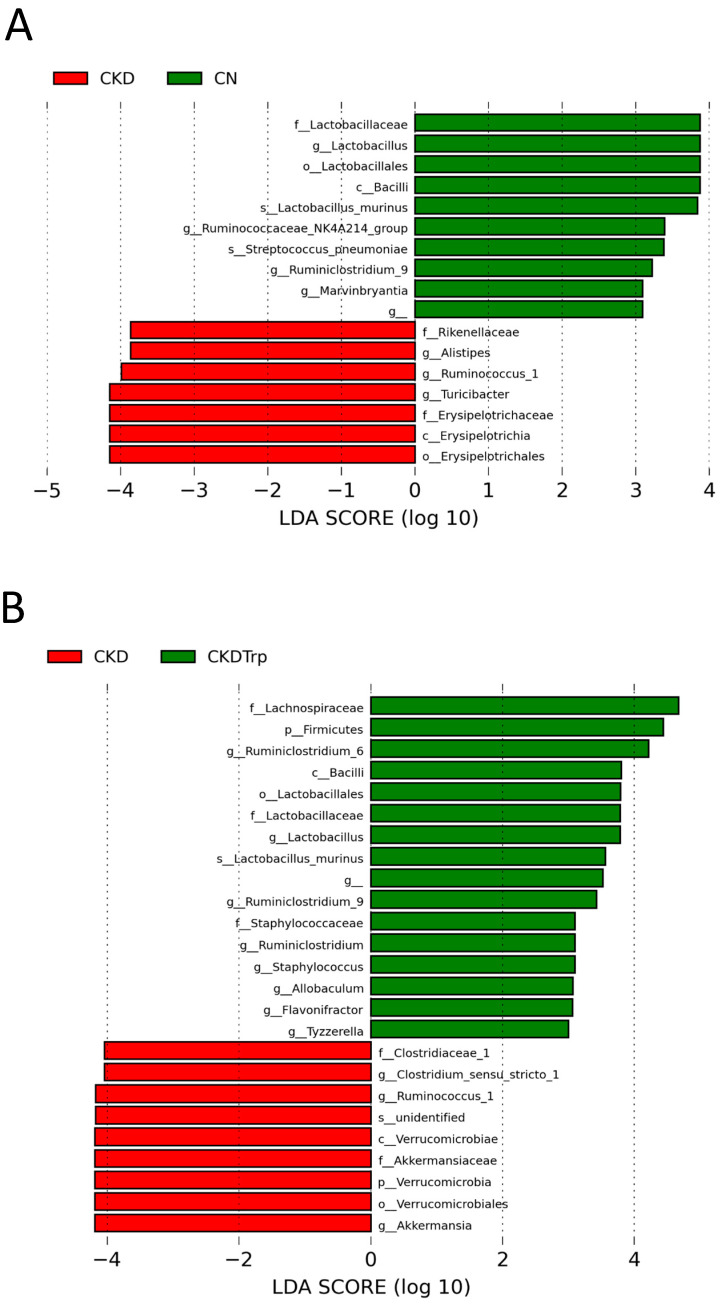
Effect of maternal adenine-induced chronic kidney disease (CKD) and tryptophan supplementation (Trp) on the gut microbiota at 12 weeks of age. Linear discriminant analysis effect size (LEfSe) was applied to identify enriched bacterial species. The threshold of the linear discriminant was set to 3. Most enriched and depleted species in the (**A**) CKD (red) versus CN group (green) and (**B**) CKD (red) versus CKDTrp group (green).

Moreover, we executed linear discriminant analysis effect size (LEfSe) algorithm to the identified metagenomics biomarker (Figure 6). A comparison between the multiple groups can be achieved by LEfSe analysis to find differentially over or under-represented taxa between the groups. The LEfSe analysis identified a higher abundance of genus *Ruminococcus_1* and lower abundance of genera *Lactobacillus* and *Ruminiclostridium_9* in the CKD group vs. the controls (Figure 6A). Additionally, CKDTrp group had a higher abundance of *Lactobacillus* and *Ruminiclostridium_9,* but a lower abundance of genera *Ruminococcus_1* and *Akkermansia* compared to the CKD group (Figure 6B; LEfSe, LDA > 3).

## 3. Discussion

Our study provides new insights into the protective roles of maternal tryptophan supplementation on hypertension programmed by maternal CKD through regulation of the gut microbiota compositions and AHR signaling pathways. The major findings in this study were: (1) maternal CKD caused the elevation of SBP in adult offspring, which maternal tryptophan supplementation prevented; (2) maternal CKD-induced offspring hypertension is related to increased plasma ADMA level and decreased l-arginine-to-ADMA ratio; (3) maternal CKD reduced RAS components *At2r*, *Ace2*, and *Mas* expression in offspring kidneys; (4) maternal CKD and tryptophan supplementation differentially shaped distinct gut microbiota profile in adult offspring; (5) maternal CKD decreased genera *Lactobacillus* and *Ruminiclostridium_9*, but increased genus *Ruminococcus_1*, which were restored by tryptophan supplementation; and (6) the protective effect of tryptophan supplementation against maternal CKD-induced programmed hypertension is associated with alterations to several tryptophan-metabolizing microbes, including *Lactobacillus*, *Ruminococcus*, *Clostridium*, and *Turicibacter*.

The present study is consistent with previous reports showing that adenine can induce CKD in adult rats [15,23,24]. Here, we found for the first time that adult offspring born to dams with adenine-induced CKD had a significant rise in SBP and the kidney weight-to-BW ratio. However, plasma creatinine level was not significantly different between the CKD and control group. These data demonstrated that maternal CKD induced renal hypertrophy and hypertension, and had no effect on renal function in 12-week-old adult male offspring.

Tryptophan and its metabolites have been shown to induce vasodilatation [25]. Of note is that the BP-lowering effect of tryptophan supplementation in pregnancy manifested from eight weeks of age onwards. These findings indicate that any reduction in the SBP after tryptophan supplementation is more likely due to a reprogramming effect than being an acute effect.

Several mechanisms have been reported involved in the developmental programming of hypertension [1,2,3]. In the current study, we provide further evidence for an association between maternal CKD-induced programed hypertension and alterations of gut microbiota compositions, decreased NO bioavailability, and reduced the nonclassical RAS axis. Previous studies have reported that animals and patients with CKD had decreases in the genera *Lactobacillus* and *Ruminiclostridium* in their gut microbiota [22,26], while the abundance of genera *Ruminococcus* and *Intestinimonas* were increased [27]. Our findings extend previous studies on the CKD-related changes of gut microbiota compositions in mother rats to their adult offspring. Additionally, the data in this work showed that maternal CKD-induced programmed hypertension is associated with NO deficiency, represented by increased plasma ADMA level and decreased L-arginine-to-ADMA ratio. Our findings are consistent with research showing that NO deficiency is a common mechanism underlying hypertension programmed by different early-life insults [16,28,29]. Another mechanism underpinning offspring hypertension programmed by maternal CKD is probably due to dysregulated RAS. We found maternal CKD reduced renal expression of *At2r*, *Ace2*, and *Mas*, which appear to be correlated with the rise of BP. This data is in agreement with the notion that lack of the protective arms of the non-classical RAS, which promote vasoconstriction and elevate BP [17]. However, these alterations in the RAS caused by maternal CKD are not normalized to control level by tryptophan supplementation. Our findings imply that mediation of the RAS might not be a major mechanism underlying the protective effect of tryptophan supplementation against maternal CKD-induced hypertension.

Several bacterial species have been reported to produce tryptophan metabolites [5,30,31], such as *Lactobacillus, Bacteroides, Bifidobacterium, Peptostreptococcus*, *Ruminococcus, Ruminiclostridium,* and *Clostridium.* Although tryptophan supplementation in pregnancy shaped a different microbiome profile compared to the controls, most tryptophan-metabolizing microbes were not altered. Interestingly, the beneficial effect of tryptophan supplementation against maternal CKD-induced programmed hypertension is associated with increased abundance of genera *Lactobacillus, Ruminiclostridium_9*, whereas the abundance of genera *Ruminococcus_1* and *Clostridium* were decreased. Since that tryptophan is a dietary uremic toxin precursor, and that alterations of several tryptophan-metabolizing microbes are related to the protective effects of tryptophan supplementation in this maternal CKD model, it is possible that these microbes can influence the specific types of tryptophan-derived metabolites in favor of BP-lowering benefit.

*Lactobacillus* has been regarded as a beneficial microbe for intestinal health in humans and animals [32]. According to our data, tryptophan supplementation restores maternal CKD-induced reduction of *Lactobacillus* abundance may be attributed to its tryptophan-metabolizing ability, to improve intestinal ecosystem and hypertension. In patients with hypertension, *Ruminiclostridium* was decreased [33] Additionally, genera *Ruminococcus* and *Clostridium* were associated with SBP [34]. These findings confirm the link between these tryptophan-metabolizing microbes and hypertension. Another tryptophan-metabolizing bacteria *Turicibacter* was in higher quantities in the spontaneously hypertensive rats (SHR) [35]. Thus, tryptophan supplementation decreased the abundance of genus *Turicibacter* might be also related to its BP-lowering benefit. Additionally, we observed that tryptophan supplementation altered the proportion of phylum *Firmicutes* and *Bacteroidetes*, thus increasing the *Firmicutes* to *Bacteroidetes* ratio. Our results contradict those previously published showing this ratio is high in animals with hypertension [22], possibly because we were experimenting in the model of developmental programming of hypertension instead of established hypertensive models.

An additional protective mechanism of tryptophan supplementation on programmed hypertension in this model may be related to mediation of the AHR signaling pathway. AHR signaling can be activated by exogenous or endogenous ligands to target AHR gene expression, such as *Cyp1a1* and *Tiparp* [36]. The AHR repressor (AHRR) is an AHR-regulated gene and a negative regulator of the AHR by competing with the AHR for the binding of the AHR nuclear translocator (ARNT) [9]. Our results demonstrated that tryptophan supplementation decreased expression of *Ahrr*, but increased expression of *Cyp1a1, Arnt*, and *Tiparp*. The present observations suggest that maternal tryptophan consumption may lead to a myriad of metabolites as endogenous AHR ligands and activate AHR signaling pathway. Although we previously reported that exogenous AHR ligand TCDD induced programmed hypertension together with activation of AHR and its target genes in adult rats [10], this notion is not supported by the present observations, which showed that tryptophan-derived endogenous AHR ligands activated AHR signaling pathway but had no effect on BP in adult offspring. This discrepancy might be due to high affinity exogenous ligands lead to AHR-associated toxicity, whereas low affinity endogenous levels of activation of AHR have been shown be beneficial [37]. On the other hand, decreased *Ahrr*, but increased *Arnt* expression in the CKDTrp group indicates AHR activation, which appears to be beneficial in BP-lowering. Since that *Lactobacillus* can convert tryptophan to indolealdehyde (IAld) and activate AHR signaling pathway [38], and that tryptophan supplementation increases *Lactobacillus* abundance and activates AHR, these findings raise the intriguing possibility that tryptophan-metabolizing microbiome may protect offspring against maternal CKD-induced hypertension via AHR. As such, whether other AHR-activating microorganisms can serve as a reprogramming strategy to prevent hypertension of developmental origins deserves additional investigation. Moreover, maternal CKD had a negligible effect on most AHR target genes, except *Ahrr.* Additional studies are required to clarify whether AHR signaling pathway plays a role in the pathogenesis of programmed hypertension in other developmental programming models.

There are some limitations that need to be taken into consideration in the current study. First, only male offspring was recruited in the present study. Whether sex differences exist in the programming effects of maternal CKD and tryptophan awaits further evaluation. Second, we did not examine tryptophan metabolites in the mother and offspring. For example, melatonin, an endogenous tryptophan metabolite, has been reported to serve as a reprogramming intervention to prevent hypertension of developmental origins [39]. Given the complex crosstalk between tryptophan metabolic pathways, the beneficial effects of maternal tryptophan supplementation come from which tryptophan metabolites deserve further clarification. Furthermore, we are well aware that the above-mentioned mechanisms in the present study might not cover the whole picture of protective role of tryptophan supplementation. Since gut microbiota derived tryptophan metabolites reported was related to inflammation and host immune status [40,41], additional studies are needed to elucidate whether these mechanisms also play a decisive role in programmed hypertension. Last, we only measure plasma creatinine level in the current study, a thorough examination of renal outcome (e.g., proteinuria, clearance of creatinine, and renal pathology) is worthy further study to determine whether adult offspring develops early stage of CKD at 12 weeks of age.

## 4. Materials and Methods

### 4.1. Animals and Experimental Design

**Figure 7:**
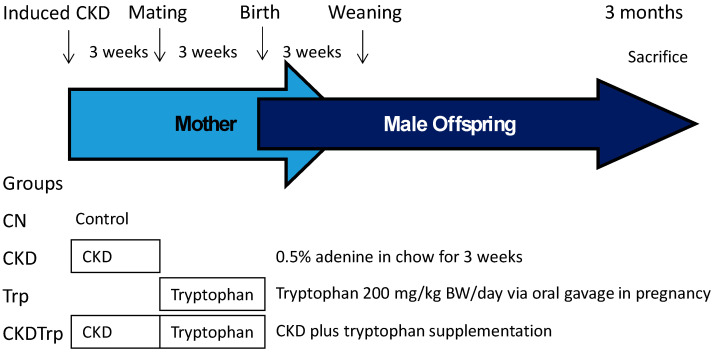
Schematic illustration of study design to establish a maternal adenine-induced chronic kidney disease (CKD) rat model to evaluate male offspring outcome at 12 weeks of age.

The experimental protocol was approved by the Institutional Animal Ethics Committee of Chang Gung Memorial Hospital (Permit Number 2019050701). All animal experiments were carried out in strict accordance with the recommendations of the Guide for the Care and Use of Laboratory Animals of the National Institutes of Health. Virgin Sprague–Dawley (SD) rats were obtained from BioLASCO Taiwan Co., Ltd. (Taipei, Taiwan) and were maintained in a core animal facility accredited by the Association for Assessment and Accreditation of Laboratory Animal Care International. The animals were exposed to light/dark cycles of 12:12 hours and controlled temperature. To construct a maternal CKD model, 8-week-old female SD rats received regular chow or chow supplemented with 0.5% adenine for 3 weeks as described previously [24]. At 11 weeks of age, some female rats (N = 5/group) were sacrificed to confirm whether they developed CKD. Rats were killed and blood was collected in heparinized tubes. According to a protocol validated in our lab [42], plasma creatinine level was determined by HPLC. The other female rats were mated with males. Adenosine-induced CKD pregnant rats received intragastric administration of either tryptophan at doses of 200 mg/kg BW/day or vehicle between 1300 and 1500 each day during pregnancy. As well, control rats received either tryptophan supplementation or vehicle during pregnancy. The dose of tryptophan used here was based on a previous study conducted in rats [43]. We only selected male offspring from each litter due to hypertension and kidney disease occur in young adulthood with higher prevalence in males than females [44,45]. Male offspring were culled to eight pups after birth, to standardize the quantity of milk and maternal pup care received, and used in subsequent experiments. Male offspring were assigned to four experimental groups (*n* = 8/group): Vehicle group (CN), adenine-induced CKD group (CKD), tryptophan supplementation group (Trp), and CKD plus tryptophan supplementation group (CKDTrp). Experimental protocol is illustrated in Figure 7. BP was measured in conscious rats at 4, 6, 8, 10, and 12 weeks of age using an indirect tail-cuff method (BP-2000; Visitech Systems, Inc., Apex, NC, USA). As we described previously [39], the rats were acclimated to restraint and tail-cuff inflation for 1 week prior to the measurement, to ensure accuracy and reproducibility. Feces from each rat were collected at 12 weeks of age. All rats were sacrificed at 12 weeks of age. Heparinized blood samples were collected at the end of the study, and the kidneys were subsequently collected. Kidneys were harvested after perfusion with phosphate buffered saline, divided into the cortex and medulla regions, snap-frozen, and stored at −80 °C freezer. Plasma creatinine level was determined by HPLC [42].

### 4.2. High Performance Liquid Chromatography (HPLC)

Plasma levels of NO-related metabolites, including l-citrulline (the precursor of l-arginine), l-arginine (substrate for NO synthesis), ADMA, and SDMA were measured using HPLC (HP series 1100; Agilent Technologies Inc., Santa Clara, CA, USA) with the OPA-3MPA derivatization reagent [42]. Homoarginine (Sigma-Aldrich, St. Louis, MO, USA) was used as the internal standard.

### 4.3. Quantitative Real-Time PCR Analysis

RNA was extracted from the kidney cortex as described previously [10,11]. Two-step quantitative real-time PCR (qPCR) was conducted using Quantitect SYBR Green PCR Reagents (Qiagen, Valencia, CA) on an iCycler iQ Multi-color Real-Time PCR Detection System (Bio-Rad, Hercules, CA). Four AHR target genes were analyzed, including *Ahrr* (encoded for aryl hydrocarbon receptor repressor), *Cyp1a1* (Cytochrome P450 CYP1A1), *Arnt* (encoded for aryl hydrocarbon receptor nuclear translocator), and *Tiparp* (encoded for TCDD-inducible poly-ADP-ribose polymerase). Additionally, we analyzed several components in the RAS, including *Agt* (angiotensinogen), *Ren* (renin), *atp6ap2* (prorenin receptor), *Ace1* (angiotensin converting enzyme-1), *Ace2*, *At1r* (angiotensin II type 1 receptor), *At2r* (angiotensin II type 2 receptor), and *Mas* (angiotensin (1-7) receptor MAS). *Rn18S* was used as a reference in all analyses. The sequences of the primers used in this study are provided in Table 3. All samples were run in duplicate. For the relative quantification of gene expression, the comparative threshold cycle (CT) method was employed. For each sample, the average C_T_ value was subtracted from the corresponding average *Rn18S* value, calculating the ΔC_T_. ΔΔC_T_ was calculated by subtracting the average control ΔC_T_ value from the average experimental ΔC_T_. The fold-increase of the experimental sample, relative to the control, was calculated using formula 2^−ΔΔCT^.

### 4.4. Metagenomics Analysis of Gut Microbiota

Frozen fecal samples were analyzed with metagenomics focused on the V3-V4 of the 16S DNA gene. As described previously [46], all polymerase chain-reaction amplicons were mixed together the Biotools Co., Ltd. (Taipei, Taiwan) for sequencing using an Illumina Miseq platform (Illumina, CA, USA). The sequences were analyzed using QIIME version 1.9.1. Sequences with a distance-based similarity of 97% or greater were grouped into operational taxonomic units (OTUs) using the USEARCH algorithm. The phylogenetic relationships were determined based on a representative sequence alignment using Fast-Tree. We compared patterns of α- and β- diversity for microbial communities [18,19]. Shannon’s diversity index accounting for both abundance and evenness of the taxa present was analyzed by QIIME version 1.9.1 [20]. We evaluated the β-diversity changes in gut microbiota across groups by the Partial Least Squares Discriminant Analysis (PLS-DA) and the Analysis of Similarities (ANOSIM) [21]. To determine the significantly differential taxa, linear discriminant analysis effect size (LEfSe) was applied to compare samples between groups. The threshold of the linear discriminant was set to 3.

### 4.5. Statistical Analysis

All data are expressed as mean ± S.E.M. Parameters were compared using two-way analysis of variance (ANOVA) followed by a Tukey’s post hoc test for multiple comparisons. Weights, plasma parameters, and SBP between the controls and CKD mother rats were analyzed by the *t*-test. A *p*-value < 0.05 was considered statistically significant. All analyses were performed using the Statistical Package for the Social Sciences software (SPSS Inc., Chicago, IL, USA).

## 5. Conclusions

In conclusion, tryptophan supplementation in pregnancy offsets the effects of maternal CKD-induced programmed hypertension, primarily related to alterations of gut microbiota compositions and the AHR signaling pathway. The RAS and NO pathways were involved in the maternal CKD-induced programmed hypertension. The fact that maternal tryptophan supplementation prevented hypertension programmed by maternal CKD potentially might be due to its effects on tryptophan-metabolizing microbes; further investigation should be warranted to evaluate which specific tryptophan-metabolizing pathways and their metabolites are related to programmed hypertension. A better understanding of the mechanisms underlying the role of tryptophan-metabolizing microbiome in the early-life programming of hypertension is essential to the development of specific reprogramming interventions to halt the worldwide epidemic of hypertension and kidney disease.

## Figures and Tables

**Table 1:** Morphological and biochemical values in different experimental groups.

Group	CN	CKD	Trp	CKDTrp		*p* Value	
Factor					CKD	Trp	CKD × Trp
Mortality	0%	0%	0%	0%			
Body weight (BW) (g)	349 ± 9	369 ± 15	350 ± 6	360 ± 10	NS	NS	NS
Left kidney weight (g)	1.43 ± 0.04	1.64 ± 0.07 *	1.55 ± 0.03	1.67 ± 0.04 *	0.001	NS	NS
Left kidney weight/100g BW	0.41 ± 0.01	0.45 ± 0.01 *	0.44 ± 0.01 *	0.47 ± 0.01 *	0.004	0.01	NS
Systolic BP (mmHg)	136 ± 1	153 ± 1 *	136 ± 1 ^#^	140 ± 1 ^#^	<0.001	<0.001	<0.001
Diastolic BP (mmHg)	70 ± 3	70 ± 2	70 ± 3	70 ± 3	NS	NS	NS
MAP (mmHg)	92 ± 2	97 ± 1 *	92 ± 1 ^#^	93 ± 2 ^#^	0.045	NS	NS
Creatinine (μM)	12 ± 0.3	12.7 ± 0.3	10 ± 0.2 *^,#^	9.9 ± 0.1 *^,#^	NS	0.001	NS

Data are shown as means ± S.E.M. A two-way ANOVA with a Tukey’s post hoc test was performed for statistical analysis. CKD × Trp = interaction of CKD and Trp. *n* = 8/group. * *p* < 0.05 vs. CN. ^#^ *p* < 0.05 vs. CKD. BP = blood pressure. MAP = mean arterial pressure. NS = not significant.

**Table 2:** Morphological and biochemical values in different experimental groups.

Group	CN	CKD	Trp	CKDTrp		*p* Value	
Factor					CKD	Trp	CKD × Trp
l-citrulline (μM)	77.4 ± 3.4	78.1 ± 1.5	64.1 ± 1 *^,#^	59.3 ± 1.8 *^,#^	NS	0.011	NS
l-arginine (μM)	154 ± 6	145 ± 4	145 ± 4	178 ± 5	NS	NS	NS
ADMA (μM)	2.38 ± 0.13	5.92 ± 0.2 *	2.85 ± 0.18 ^#^	5.52 ± 0.18 *	<0.001	NS	NS
SDMA (μM)	2.45 ± 0.09	2.24 ± 0.03	1.84 ± 0.04 *^,#^	1.99 ± 0.04 *^,#^	NS	0.011	NS
AAR (μM/μM)	72 ± 3	26 ± 1 *	63 ± 5 ^#^	35 ± 2 *	<0.001	NS	NS

Data are shown as means ± S.E.M. A two-way ANOVA with a Tukey’s post hoc test was performed for statistical analysis. CKD × Trp = interaction of CKD and Trp. *n* = 8/group. * *p* < 0.05 vs. CN. ^#^ *p* < 0.05 vs. CKD. ADMA = asymmetric dimethylarginine. SDMA = symmetric dimethylarginine. AAR = l-arginine-to-ADMA ratio. NS = not significant.

**Table 3:** Quantitative real-time polymerase chain reaction primers sequences.

Gene	Forward (5′–3′)	Reverse (5′–3′)
*Ahrr*	cagcaacatggcttctttca	tgaagcactgcattccagac
*Cyp1a1*	gcactctggacaaacacctg	atatccaccttctcgcctgg
*Arnt*	gtctccctcccagatgatga	gctggtagccaacagtagcc
*Tiparp*	gttgagggccaattaccaga	gctcctggcacataatccat
*Ren*	aacattaccagggcaactttcact	acccccttcatggtgatctg
*Atp6ap2*	gaggcagtgaccctcaacat	ccctcctcacacaacaaggt
*Agt*	gcccaggtcgcgatgat	tgtacaagatgctgagtgaggcaa
*Ace1*	caccggcaaggtctgctt	cttggcatagtttcgtgaggaa
*Ace2*	acccttcttacatcagccctactg	tgtccaaaacctaccccacatat
*At1r*	gctgggcaacgagtttgtct	cagtccttcagctggatcttca
*At2r*	caatctggctgtggctgactt	tgcacatcacaggtccaaaga
*Mas*	catctctcctctcggctttgtg	cctcatccggaagcaaagg
*Rn18S*	gccgcggtaattccagctcca	cccgcccgctcccaagatc

## Abbreviations

ACE	Angiotensin converting enzyme
ACE2	Angiotensin converting enzyme 2
ADMA	Asymmetric dimethylarginine
AHR	Aryl hydrocarbon receptor
ANOSIM	Analysis of Similarities
AT1R	Angiotensin II type 1 receptor
AT2R	Angiotensin II type 2 receptor
CKD	Chronic kidney disease
LDA	Linear discriminant analysis
LEfSe	Linear discriminant analysis effect size
MAS	Angiotensin (1-7) receptor
PLS-DA	Partial Least Squares Discriminant Analysis
RAS	Renin–angiotensin system
SD	Sprague–Dawley
SDMA	Symmetric dimethylarginine
